# Diagnostic value and integrated threshold of ESR for diabetic foot osteomyelitis: a systemic review and meta-analysis

**DOI:** 10.3389/fendo.2025.1660465

**Published:** 2025-09-25

**Authors:** Lulu Liu, Yukun Tao, Da Zhang, Jinzheng Hou, Guangxin Zhou, Maolin Tian

**Affiliations:** ^1^ Department of Endocrinology, Air Force Medical Center, Air Force Medical University, Beijing, China; ^2^ Hebei North University, Zhangjiakou, Hebei, China

**Keywords:** diabetic foot osteomyelitis, erythrocyte sedimentation rate, diagnostic accuracy, meta-analysis, hierarchical summary ROC curve, diagnostic cutoff, inflammatory biomarkers

## Abstract

**Background:**

Diabetic foot osteomyelitis (DFO) is a severe complication of diabetic foot infections (DFI). Early and accurate diagnosis is crucial for improving patient outcomes. The erythrocyte sedimentation rate (ESR), a commonly used inflammatory marker, has been controversial regarding its diagnostic value and optimal cutoff values in DFO.

**Objective:**

This study aims to conduct a systematic review and meta-analysis to comprehensively evaluate the diagnostic efficacy of ESR for DFO and to determine an optimal pooled cutoff value for the marker.

**Methods:**

A systematic search of PubMed, EMBASE, Cochrane Library, OVID, and the Wanfang database was conducted for literature related to ESR in the diagnosis of DFO, with the search period extending through March 2025. The Quality Assessment of Diagnostic Accuracy Studies-2 (QUADAS-2) tool was applied for quality evaluation of the included studies. Statistical analyses were performed using Stata 18.0 to generate hierarchical summary receiver operating characteristic (HSROC) curves and forest plots for assessing the diagnostic performance of ESR in DFO. To determine the optimal composite cutoff value of ESR for diagnosing DFO, a different random intercepts and common random slope (DICS) model was implemented using R4.5.0. Subsequently, a Generalized Linear Mixed Model (GLMM) was constructed to predict the corresponding sensitivity and specificity at the ESR threshold of 70 mm/h.

**Results:**

Following the Preferred Reporting Items for Systematic Reviews and Meta-Analyses (PRISMA) guidelines, 12 studies with a total of 1,674 subjects were included. The HSROC model revealed that the area under the curve (AUC) for ESR in diagnosing DFO was 0.71, with sensitivity and specificity values of 0.76 and 0.73, respectively. The DICS model identified an optimal pooled cutoff value for ESR at 51.6 mm/h, with corresponding sensitivity and specificity values of 0.80 and 0.67, respectively. Using the GLM model, an ESR cutoff of 70 mm/h yielded sensitivity and specificity of 0.61 and 0.83, respectively.

**Conclusion:**

ESR demonstrates moderate diagnostic efficacy in the identification of DFO. Based on our findings, we recommend the optimal pooled cutoff value for ESR is 51.6 mm/h, as a preliminary screening tool in the diagnostic workup of DFO.

## 1 Introduction

diabetic foot infections(DFI) is one of the most common reasons for hospitalization among patients with diabetes (1). Approximately 20% of patients with DFI and 50% of those with severe DFI may progress to Diabetic foot osteomyelitis(DFO) (2), a condition strongly associated with an increased risk of amputation (3). Timely diagnosis of DFO and the initiation of effective antibiotic therapy have the potential to mitigate the risk of amputation caused by DFO (4). Consequently, distinguishing between soft tissue infection and osteomyelitis is of paramount importance.

Erythrocyte sedimentation rate(ESR) is routinely utilized in clinical practice to detect acute inflammatory responses and monitor inflammatory status. As a slow-reacting acute-phase reactant, ESR exhibits an initial elevation occurring 24 to 48 hours after the onset of inflammation and declines gradually following its resolution (5, 6). The diagnostic value of ESR for DFO has been the focus of several meta-analyses. Majeed et al. (7), Sharma et al. (8), and Ansert et al. (9) have conducted meta-analyses evaluating the diagnostic utility of ESR for DFO. These studies employed conventional receiver operating characteristic (ROC) curve analysis to calculate the area under the curve (AUC) and explored composite ESR cutoff values using methods such as identifying the mode or calculating the mean.

However, conventional ROC curve analysis for AUC estimation does not incorporate all reported cutoff values along with their corresponding sensitivities and specificities from the included studies. Consequently, the results may be influenced by heterogeneity among individual cutoff points. Furthermore, calculating a composite cutoff using the mean or mode fails to account for variations between different cutoffs and the impact of outliers, potentially compromising diagnostic accuracy.The Cochrane Library recommends the hierarchical summary receiver operating characteristic (HSROC) model (10) for evaluating the overall diagnostic performance in diagnostic meta-analyses. The HSROC model, grounded in Bayesian statistics, incorporates the sensitivity and specificity associated with each cutoff value reported across studies to construct the model, thereby deriving the AUC along with summary estimates of sensitivity and specificity. The optimal composite cutoff value is calculated using the different random intercepts and common random slope (DICS) model. The DICS model (11, 12) is a linear mixed-effects model that integrates multiple cutoff values and their corresponding sensitivities and specificities from different diagnostic studies into a continuous distribution model. It subsequently determines the optimal composite cutoff value by maximizing the Youden index.

This study aims to synthesize the available research on ESR for DFO diagnosis through a meta-analysis to elucidate its diagnostic performance. Specifically, we will employ the HSROC model to estimate the diagnostic value of ESR for DFO and utilize the DICS model to identify the optimal composite cutoff value along with its corresponding sensitivity and specificity.

## 2 Methods

### 2.1 Data sources and search strategy

Following the Preferred Reporting Items for Systematic Reviews and Meta-Analyses (PRISMA) guidelines (13), the following procedure was implemented: identifying relevant studies, conducting an initial screening, performing an in-depth screening, extracting data, evaluating the quality of the included studies, and conducting a meta-analysis. A comprehensive literature search was conducted across five databases, including PubMed, EMBASE, Cochrane Library, OVID, and WanFang. The search was performed up to March 2025. The search strategy combined controlled vocabulary (MeSH terms) and free text, utilizing keywords such as “diabetic foot,” “osteomyelitis,” “inflammatory marker,” and “erythrocyte sedimentation rate.” The complete search strategy is provided in **Appendix 1**. To ensure the comprehensiveness of the study, citation tracking of eligible studies was also performed to identify relevant research potentially overlooked during the database search.

### 2.2 Study selection

The screening of studies was independently conducted by two researchers (Lulu Liu and Yukun Tao) to ensure scientific rigor and consistency. The selection process comprised three stages: initially, duplicate records were identified and removed using the reference management software EndNote X9. Subsequently, the titles, abstracts, and methodologies of the remaining articles were screened by the researchers to exclude those that did not meet the inclusion criteria. Finally, the full-text articles were thoroughly reviewed and assessed for eligibility. The inclusion criteria were as follows: No language restrictions were applied, and only studies with accessible full texts were included. The study population comprised patients aged ≥18 years with diabetic foot infections. The gold standard for diagnosing DFO was defined as histopathology, bone culture, or imaging methods (MRI/X-ray). Studies were required to report cutoff values and related diagnostic metrics for ESR in diagnosing DFO (e.g., AUC, sensitivity, and specificity).

The exclusion criteria were as follows: Case reports, reviews, systematic reviews, commentaries, and animal studies. Studies with overlapping data or insufficient diagnostic parameters for extraction. Studies with sample sizes <10 cases.

### 2.3 Data extraction and quality assessment

Following the preliminary screening, the full texts of the eligible studies were reviewed, and data were extracted. The following steps were performed to ensure rigor and accuracy: two researchers (Lulu Liu and Yukun Tao) independently evaluated the studies based on the inclusion criteria. Discrepancies during the review were resolved through discussion to achieve a consensus. Extracted data included key information, such as author names and publication years, total sample sizes, the number of DFO patients, the gold standard for diagnosing osteomyelitis, the reported ESR cutoff values, and corresponding diagnostic performance metrics (e.g., sensitivity, specificity, positive predictive value, negative predictive value, and AUC. For studies that diagnostic parameters such as true positives (TP), false positives (FP), true negatives (TN), and false negatives (FN) were not directly available, these values were calculated whenever possible based on the information provided in the original publications.

The quality of the included studies was assessed using the Quality Assessment of Diagnostic Accuracy Studies-2 (QUADAS-2) tool (14, 15), a widely recognized evidence-based tool in diagnostic research for evaluating the risk of bias. The quality assessment was performed independently by the two researchers (Lulu Liu and Yukun Tao), and disagreements were resolved through consensus to finalize the quality rating of each study.

### 2.4 Data analysis

Data from all included studies were pooled to extract ESR cutoff values and corresponding TP, FP, TN, and FN metrics. The analysis was conducted using Stata 18.0 software. The “metandi” command was utilized to generate the HSROC curve, while the “midas” command was used to create forest plots to comprehensively evaluate the diagnostic performance of ESR for DFO. Additionally, the “digmeta” package in R version 4.5.0 was employed to construct a DICS model, which identified the optimal unified cutoff value by maximizing the Youden index.

Furthermore, the Generalized Linear Mixed Model (GLMM) (16, 17) was implemented using the “lme4” package in R to predict sensitivity and specificity across different cutoff values by considering continuous cut-point modeling and random-effect stratification. The sensitivity and specificity corresponding to an ESR value of 70 mm/h which was recommended as a cutoff value to screen for DFO by International Working Group on the Diabetic Foot (IWGDF), were calculated based on the GLMM results.

### 2.5 Sensitivity analysis

Sensitivity analysis was performed on all included studies using the “metaninf” command in Stata 18.0. Studies identified as outliers based on markedly deviating effect estimates were removed, and the same analysis was repeated on the remaining studies. The absence of significant outliers in the subsequent analysis indicates that the overall results of the meta-analysis are robust.

## 3 Results

**Figure 1:**
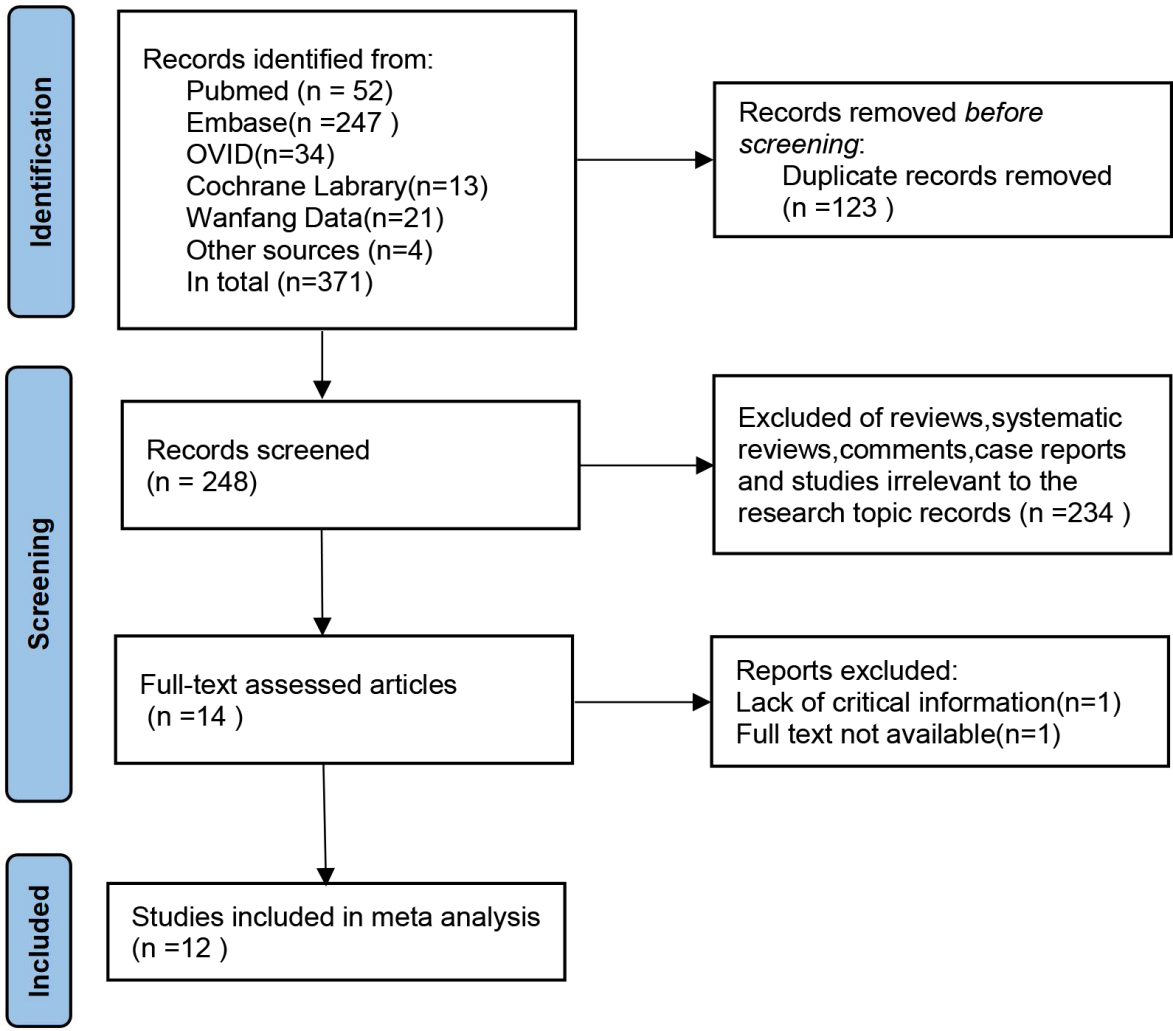
Preferred reporting items for meta-analyses flow diagram of study selection.

In the preliminary search, 371 studies were identified. After deduplication, irrelevant articles such as systematic reviews, review papers, commentaries, case reports, and studies unrelated to the research topic were excluded based on title and abstract screening. Fourteen studies progressed to the full-text review stage. After comprehensive evaluation, 12 studies (18–29) with a total sample size of 1,674 cases were ultimately included in the systematic review and meta-analysis (**Figure 1**; **Table 1**).

**Table 1:** Characteristics of studies included in the meta-analysis.

Author	Year	Country	Number of participants(n=1674)		Age	DFO (n=813)	NDFO (n=861)	Prevalence of DFO		Gold standard for the diagnosis of osteomyelitis
Crisologo (20)	2020	USA	35		NA	24	11	69%		Bone histopathology/bone culture
Fleischer (27)	2009	Chicago	54		61.5(27,90)	34	20	63%		Bone histopathology
Hadavand (23)	2019	Iran	200		61.26 ± 11.32	72	128	36%		MRI
kaleta (22)	2001	USA	29		NA	19	10	66%		Bone histopathology
Lavery (18)	2019	USA	353		54(46,61)	176	177	50%		Bone histopathology/bone culture
Michail (19)	2013	Greece	61		63.1 ± 7.1	27	34	44%		Positive probe-to-bone test+X ray/Scintillation phenomenon/MRI
Moallemi (26)	2020	Iran	142		61.2 ± 11.8	71	71	50%		X ray/MRI
Mutluog (28)	2011	Turkey	24		61.9 ± 10.8	13	11	54%		MRI
Ozer Balin (29)	2022	Turkey	247		NA	96	151	39%		Bone histopathology/bone culture
Soleimani (25)	2021	Iran	90		NA	45	45	50%		MRI
xu (24)	2021	China	197		NA	111	86	56%		Bone histopathology/bone culture
Coye (21)	2023	USA	242		53 (45,60)	125	117	52%		Bone histopathology/bone culture

Author	Year	Country	Cutoff (mm/h)	Sensitivity	Specificity	AUC	TP	FP	FN	TN
Crisologo (20)	2020	USA	73.5 *	0.63	1	0.78	15	0	9	11
Fleischer (27)	2009	Chicago	60*	0.68	0.70	0.88	23	6	11	14
Hadavand (23)	2019	Iran	56.5*	0.96	0.50	0.87	69	64	3	64
kaleta (22)	2001	USA	60	0.90	0.90	NA	17	1	2	9
			65	0.90	0.90	NA	17	1	2	9
			70*	0.90	1	NA	17	0	2	10
			75	0.84	1	NA	16	0	3	10
			80	0.79	1	NA	15	0	4	10
Lavery (18)	2019	USA	20	0.98	0.13	NA	172	154	4	23
			30	0.97	0.25	NA	171	133	5	44
			40	0.89	0.34	NA	157	117	19	60
			50	0.82	0.43	NA	144	101	32	76
			60*	0.73	0.56	NA	128	78	48	99
			70	0.60	0.65	NA	106	62	70	115
			80	0.54	0.72	NA	95	50	81	127
			90	0.46	0.78	NA	8	39	95	138
Michail (19)	2013	Greece	30	0.92	0.16	NA	25	29	2	5
			50	0.88	0.47	NA	24	18	3	16
			67*	0.84	0.75	NA	23	9	4	25
			70	0.61	0.79	NA	16	7	11	27
Moallemi (26)	2020	Iran	49*	0.75	0.58	0.70	53	30	18	41
Mutluog (28)	2011	Turkey	47*	0.73	0.85	0.74	9	2	4	9
Ozer Balin (29)	2022	Turkey	74.5*	0.63	0.73	0.72	60	41	36	110
Soleimani (25)	2021	Iran	44.5	0.89	0.80	NA	40	9	5	36
			47.5	0.87	0.84	NA	39	7	6	38
			53.5*	0.84	0.91	NA	38	4	7	41
xu (24)	2021	China	43*	0.83	0.71	0.83	92	25	19	61
			50	0.48	0.89	NA	53	9	58	77
Coye (21)	2023	USA	61*	0.71	0.61	0.71	89	46	36	71

*The best cutoff value for this study.

These studies were geographically diverse, originating from the United States (n=4), Iran (n=3), Turkey (n=2), as well as Greece and China. Sample sizes ranged from 24 to 353 patients. All studies recruited patients with DFI, of which four specifically focused on moderate-to-severe infections.

Five studies used histopathological analysis or bone culture as the reference method for diagnosing DFO, while four utilized magnetic resonance imaging (MRI) as the diagnostic criterion. Additionally, three studies employed a combination of X-ray imaging, probe-to-bone tests (PTB), or radionuclide scintigraphy as diagnostic methods. Nine studies explicitly excluded individuals with other infectious diseases or comorbidities that could affect ESR levels, such as uncontrolled rheumatic disorders, active infections, anemia, or chronic kidney disease.

**Figure 2:**
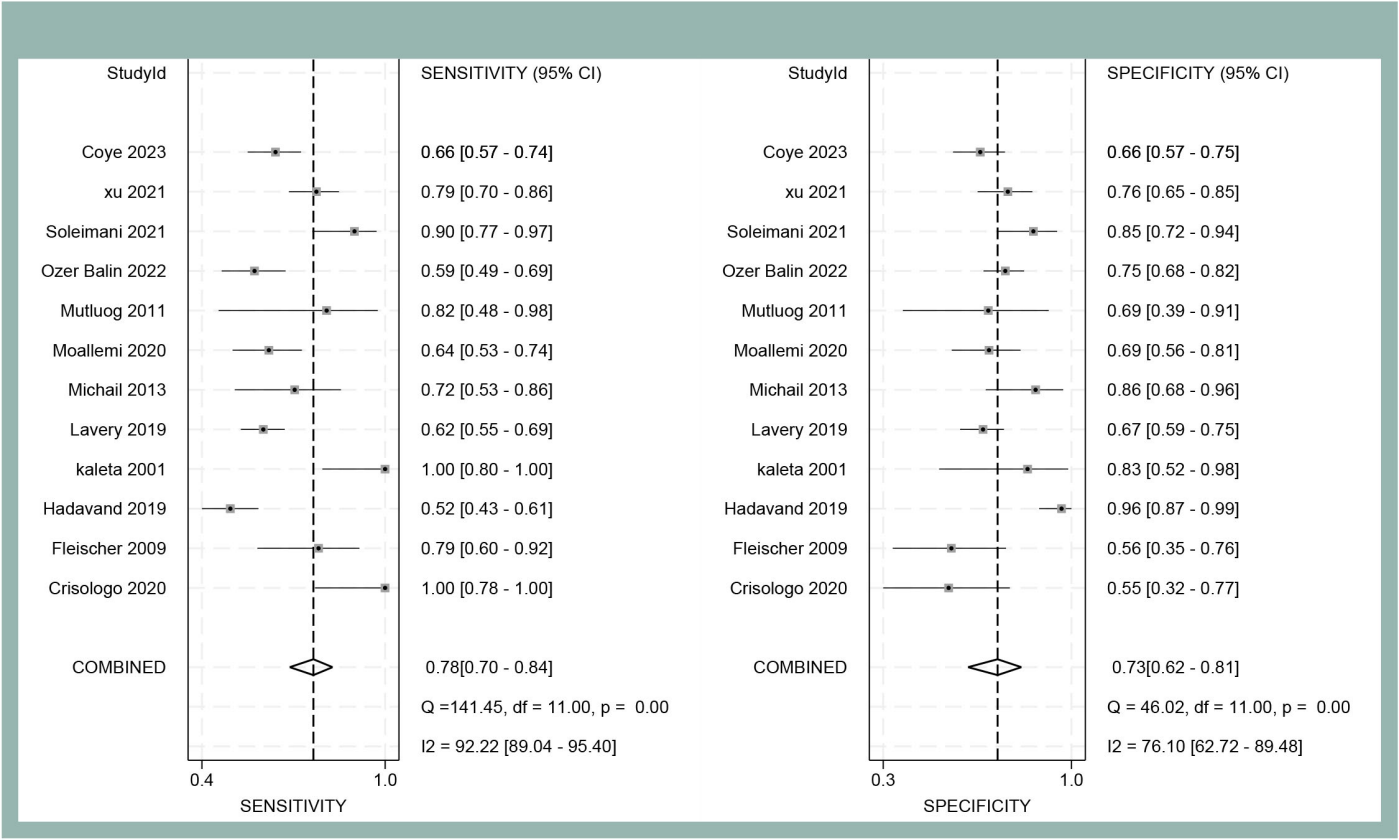
Forest plot of sensitivity and specificity of ESR for diagnosing DFO at the optimal cut-off value in the meta-analysis.

**Figure 3:**
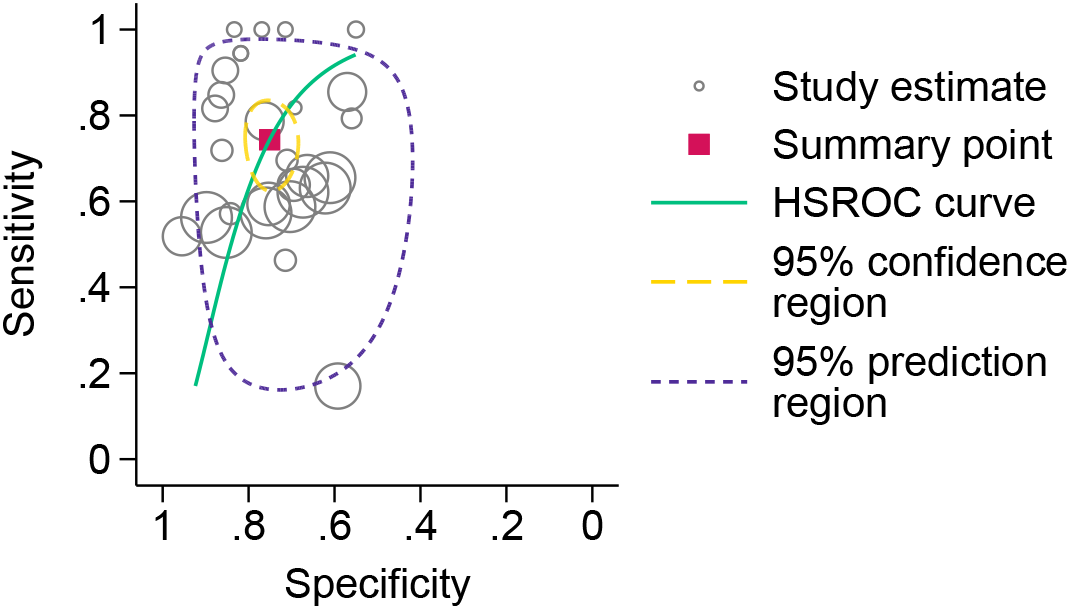
HSROC model for the diagnosis of osteomyelitis in diabetic foot using erythrocyte sedimentation rate.

The forest plot analysis (**Figure 2**) revealed the pooled sensitivity and specificity of ESR in diagnosing DFO to be 0.78 (95% CI: 0.70–0.84) and 0.73 (95% CI: 0.62–0.81), respectively. The heterogeneity statistics indicated high variability, with sensitivity I²=92.22% and specificity I²=76.10%. The HSROC model (**Figure 3**) demonstrated the AUC for ESR in diagnosing DFO as 0.71, with a pooled sensitivity of 0.76 and specificity of 0.73.

**Figure 4:**
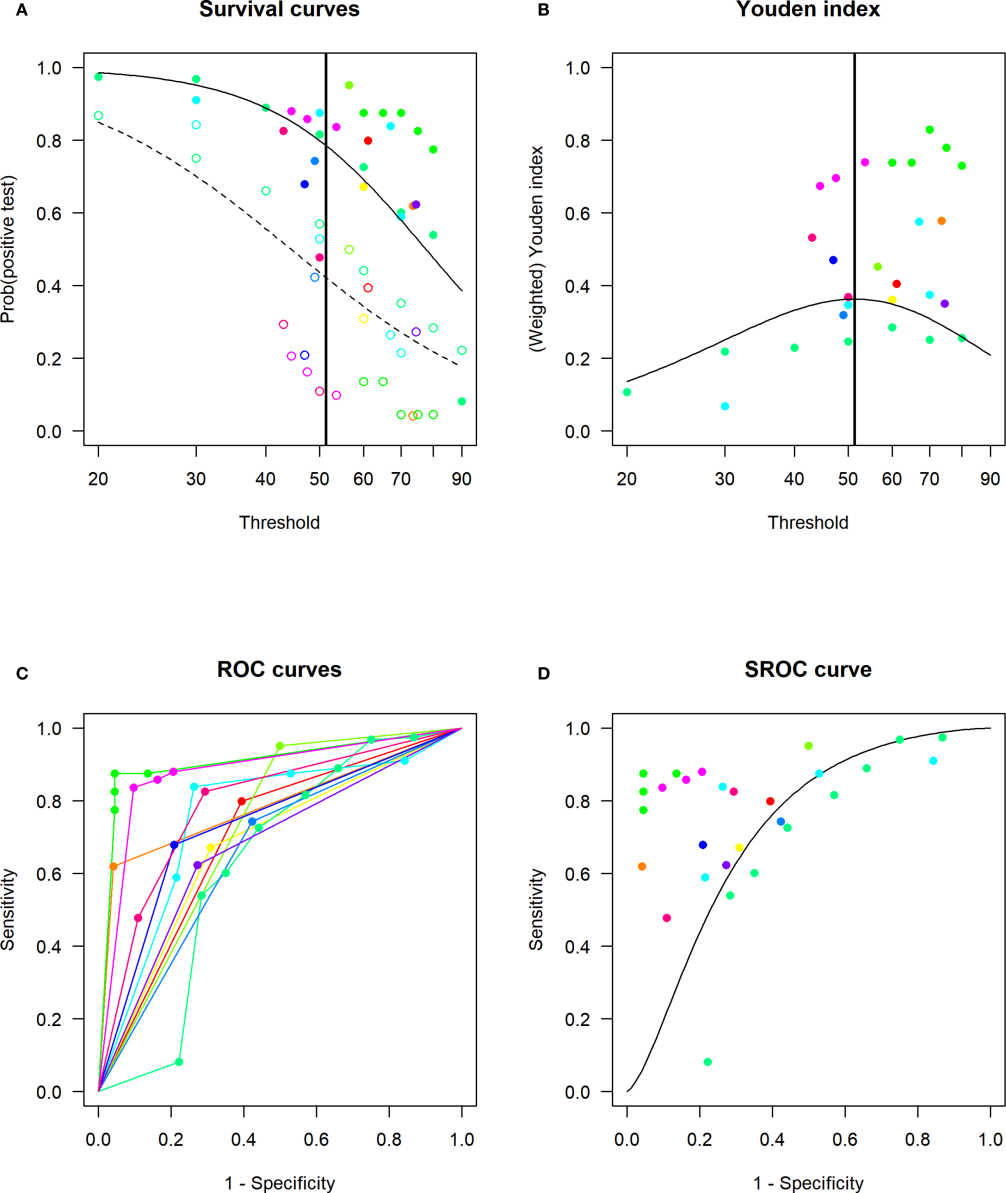
Linear mixed-effects model (DICS model) for determining the optimal cut-off value of ESR for DFO and assessing diagnostic accuracy. **(A)** Estimated distribution functions for the non-DFO (open circles, dashed line) and the DFO (filled circles, solid line). The grey lines mark the confidence regions and different studies are marked in different colours. The optimal threshold, derived from a maximization of the Youden index, is depicted as a solid vertical line. **(B)** Estimated densities and their point of intersection. Non-DFO (dashed line), DFO (solid line). **(C)** Study-specific ROC curves. **(D)** Estimated SROC curve with the optimal thresholds for different weightings of sensitivity and specificity marked as crosses in black, red and green. Different studies are marked in different colours.

The DICS model (**Figure 4**) identified the optimal unified cutoff value for ESR in diagnosing DFO as 51.6 mm/h, corresponding to a sensitivity of 0.80 and a specificity of 0.67. When ESR was set at 70 mm/h, the sensitivity decreased to 0.61, while specificity increased to 0.83.

**Figure 5:**
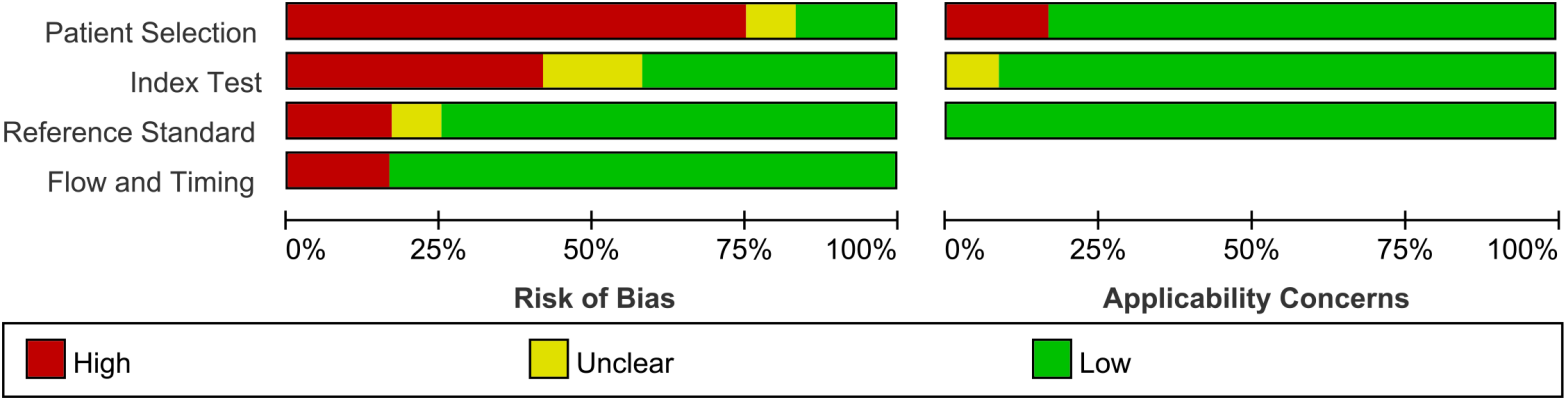
QUADAS 2 assessment for all 12 included studies by question.

The bias risk of each included study was evaluated using the QUADAS-2 tool (**Figure 5**). The proportion of studies assessed as low risk varied across domains: approximately 16% (n=2) in patient selection, 41% (n=5) in the index test, 75% (n=9) in the reference standard and 83% (n=10) in the flow and timing criteria.

The sensitivity analysis demonstrated that the pooled estimate remained stable regardless of which individual study was excluded, with values ranging from 2.14 to 3.33. Moreover, all recalculated confidence intervals retained statistical significance, indicating that the results of the meta-analysis are robust and not unduly influenced by any single study. These findings collectively confirm the reliability and stability of the meta-analytic outcomes.The results of the sensitivity analysis are presented in **Supplementary Figure 1**.

## 4 Discussion

This meta-analysis evaluated the diagnostic performance of the ESR for detecting DFO and estimated its optimal unified cutoff value. The HSROC curve demonstrated moderate diagnostic accuracy of ESR for DFO, with AUC of 0.71, a sensitivity of 0.76, and a specificity of 0.73. The optimal unified cutoff value was identified as 51.6 mm/h, with a sensitivity of 0.80 and a specificity of 0.67. We recommend 51.6 mm/h as the preliminary screening threshold for diagnosing DFO.

In 2019, Majeed et al. (7) synthesized findings from 6 studies published up to December 2017 investigating ESR for DFO diagnosis. Their meta-analysis yielded an AUC of 0.84 for ESR, with a pooled sensitivity of 0.81 and specificity of 0.80. Using the mode of the reported cutoff values across these six studies, they proposed ESR ≥70 mm/h as the diagnostic cutoff. This value (ESR ≥70 mm/h) was subsequently adopted as the diagnostic cutoff for DFO in the IWGDF(2023) guidelines (30). More recently, Sharma et al. (8) and Ansert et al. (9) have also evaluated the diagnostic value and optimal cutoff for ESR in DFO. Sharma et al., incorporating 5 studies involving 780 patients with grade 2 and 3 DFI, reported a mean ESR cutoff of 55.9 mm/h, an AUC of 0.80, and pooled sensitivity and specificity of 0.80 and 0.57, respectively. Ansert et al., analyzing 12 studies encompassing 1693 diabetic patients with soft tissue and/or bone infections, identified a mean ESR cutoff of 57.6 mm/h, an AUC of 0.83, and pooled sensitivity and specificity of 0.70 and 0.77, respectively. As outlined in the introduction, the methodologies employed in these meta-analyses for calculating AUC and composite cutoffs possess inherent limitations.

In the present study, to investigate the diagnostic accuracy of ESR for DFO, we employed the HSROC model. This model incorporates all reported cutoff values along with their corresponding sensitivities and specificities from every included study. Utilizing hierarchical Bayesian random-effects modeling, it comprehensively accounts for variation at multiple levels. The model provides an intuitive visualization of the HSROC curve with its 95% confidence interval and 95% prediction region, offering advantages over traditional summary ROC approaches. For determining the composite cutoff value, we implemented the DICS model. DICS, a linear mixed-effects model, performs parameter estimation on log-transformed data. It synthesizes all cutoff values from the 12 included studies, accommodating variations in the number of cutoffs reported per study. This model integrates all reported cutoffs and their associated sensitivities and specificities, overcoming the limitation of traditional bivariate models requiring a single cutoff per study, thereby enhancing the reliability of our findings.

Using the DICS model, we identified an optimal composite ESR cutoff for DFO diagnosis of 51.6 mm/h, demonstrating a notable discrepancy from the guideline-recommended cutoff of ≥70 mm/h. Consequently, we constructed GLMM to incorporating all reported cutoffs, sensitivities, and specificities from the included literature to predict the diagnostic performance at ESR = 70 mm/h. This yielded a sensitivity of 0.61 and a specificity of 0.83. This lower sensitivity suggests that the ≥70 mm/h cutoff risks missed diagnoses of DFO in some patients, potentially leading to delayed treatment, prolonged wound healing, and an increased amputation risk. However, it is crucial to note that our composite cutoff (51.6 mm/h) exhibited a specificity of only 0.69. Therefore, in clinical practice, ESR results must be interpreted in conjunction with the patient’s history, clinical presentation, and other ancillary investigations. When feasible, definitive diagnosis of DFO should be confirmed by bone culture or histopathology. We propose our composite ESR cutoff as an initial screening tool for DFO.

Despite the observed heterogeneity in our meta-analysis, the diagnostic performance of ESR for DFO, as determined by our methodology, demonstrates comparable results to the three previously published reviews on this topic. Based on the AUC findings, all analyses consistently position ESR as possessing moderate diagnostic value for DFO.

## 5 Limitations

However, our study has several limitations. First, significant heterogeneity was observed among the studies included in this meta-analysis. Although all study populations comprised DFI patients, the severity of infection varied considerably. Fleischer et al. (27) exclusively included patients with mild-to-moderate foot infections and acute osteomyelitis, excluding those with chronic osteomyelitis, while four other studies focused on moderate-to-severe DFI patients. The DFO prevalence rates also differed based on infection severity, potentially influencing diagnostic efficacy of ESR for DFO. And some investigations did not exclude subjects with concurrent conditions such as acute infections, anemia, or azotemia, which may have influenced baseline ESR measurements. Second, the reference standards for diagnosing DFO were inconsistent. Five studies relied on imaging (X-ray or MRI) rather than the definitive standards of bone histopathology or culture. While imaging may accurately identify chronic osteomyelitis, it might fail to detect early, mild osteomyelitis cases that could be missed by X-ray or probe-to-bone testing. When MRI is employed as the reference standard, the presence of non-infectious pathologies—such as trauma, prior foot surgery, or reactive bone marrow edema associated with Charcot neuropathic osteoarthropathy—can diminish its specificity and positive predictive value. These factors may introduce diagnostic bias for DFO. Finally, we did not explore ESR cutoff values stratified by varying degrees of infection severity. These findings underscore the need for future prospective, multicenter studies involving more diverse populations to establish more universally applicable cut-off values for ESR. It is also essential to standardize patient selection and reference standards to minimize bias introduced by heterogeneity in study cohorts and diagnostic criteria. Furthermore, a stratified analysis of ESR cut-off values should be performed according to the severity of infection in patients.

## 6 Conclusion

This study evaluates the diagnostic value of ESR in detecting DFO through a meta-analysis, providing a comprehensive cutoff value. The results of the meta-analysis indicate that ESR demonstrates moderate diagnostic efficacy for DFO and exhibits relatively high sensitivity. We recommend an ESR cutoff value of 51.6 mm/h as the optimal threshold for the preliminary screening of DFO.

## Data Availability

The original contributions presented in the study are included in the article/**Supplementary Material**. Further inquiries can be directed to the corresponding author.

## Supplementary material

Supplementary Figure 1 Sensitivity Analysis for the Pooled Sensitivity and Specificity. The figure displays the meta-analysis estimates (with 95% CI) obtained by omitting each study one at a time. The consistent stability of both the point estimates and confidence intervals demonstrates that no single study disproportionately influenced the overall results.
