# Supplementary figures and images for: Diagnostic value and integrated threshold of ESR for diabetic foot osteomyelitis: a systemic review and meta-analysis
Source: Front Endocrinol (Lausanne). 2025 Sep 25;16:1660465. doi: 10.3389/fendo.2025.1660465 (PMC12507639; doi:10.3389/fendo.2025.1660465)

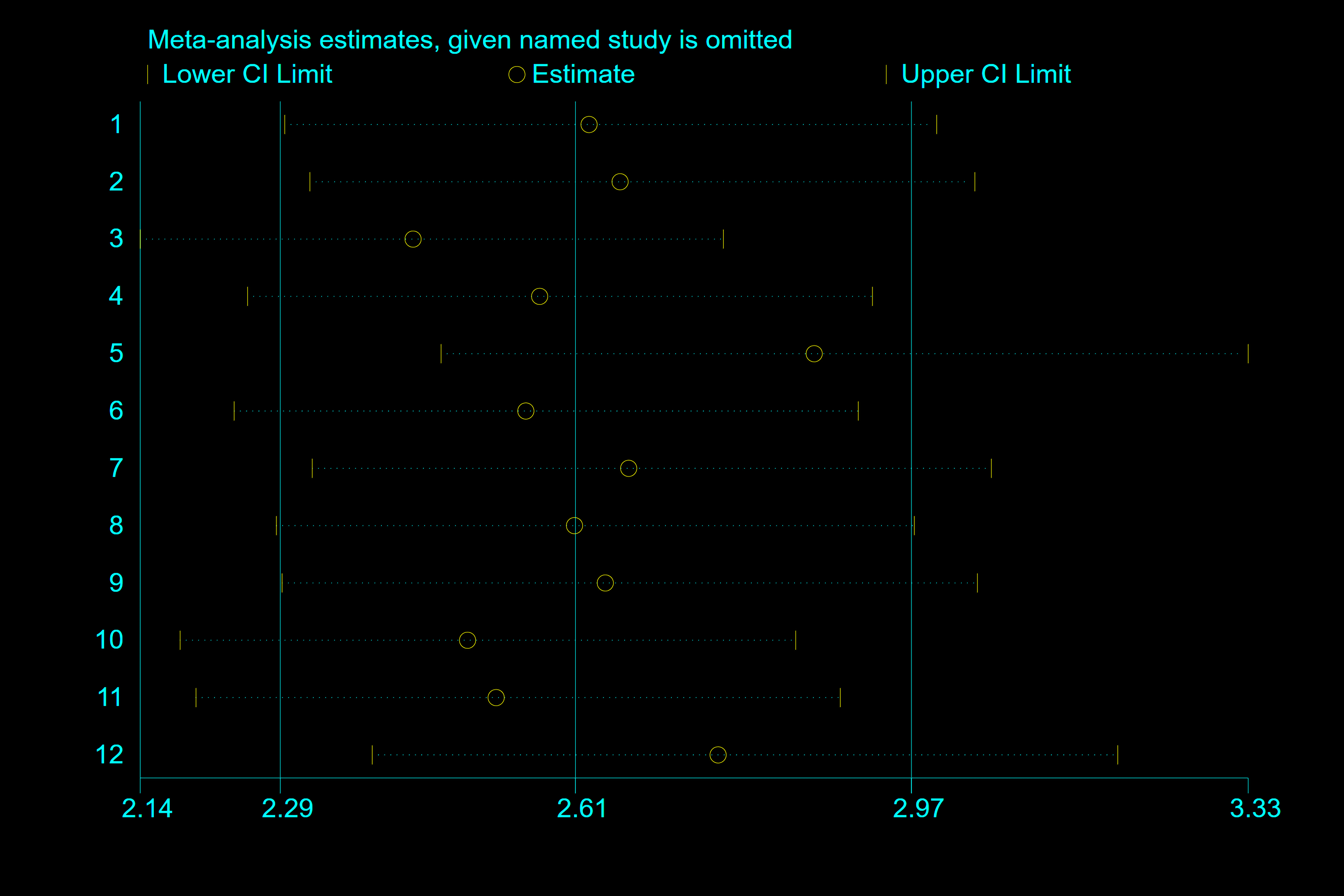

Supplement: Supplementary Figure 1 — Sensitivity Analysis for the Pooled Sensitivity and Specificity. The figure displays the meta-analysis estimates (with 95% CI) obtained by omitting each study one at a time. The consistent stability of both the point estimates and confidence intervals demonstrates that no single study disproportionately influenced the overall results. [file Image1.tif]
