# Supplementary material for: Diagnostic value and integrated threshold of ESR for diabetic foot osteomyelitis: a systemic review and meta-analysis
Source: Front Endocrinol (Lausanne). 2025 Sep 25;16:1660465. doi: 10.3389/fendo.2025.1660465 (PMC12507639; doi:10.3389/fendo.2025.1660465)
Supplement: Supplementary file 2 [file DataSheet1.docx]

**search strategy**

**Pumed: 52**

Search: **(("Osteomyelitis"[Mesh]) AND (("Diabetic Foot"[Mesh]) OR ((((Foot, Diabetic[Title/Abstract]) OR (Diabetic Feet[Title/Abstract])) OR (Feet, Diabetic[Title/Abstract])) OR (Foot Ulcer, Diabetic[Title/Abstract])))) AND (("Blood Sedimentation"[Mesh]) OR ((((((((((((Sedimentation, Blood[Title/Abstract]) OR (Erythrocyte Sedimentation[Title/Abstract])) OR (Sedimentation, Erythrocyte[Title/Abstract])) OR (Erythrocyte Sedimentation Rate[Title/Abstract])) OR (Erythrocyte Sedimentation Rates[Title/Abstract])) OR (Rate, Erythrocyte Sedimentation[Title/Abstract])) OR (Rates, Erythrocyte Sedimentation[Title/Abstract])) OR (Sedimentation Rate, Erythrocyte[Title/Abstract])) OR (Sedimentation Rates, Erythrocyte[Title/Abstract])) OR (ESR[Title/Abstract])) OR (inflammatory marker[Title/Abstract])) OR (inflammatory markers[Title/Abstract])))**

****Embase: 247****

**Embase**

**Session Results**

**.......................................................**

**No. Query Results Results Date**

**#10. #7 AND #8 AND #9 247 11 Feb 2025**

**#9. #5 OR #6 62,850 11 Feb 2025**

**#8. #3 OR #4 170,122 11 Feb 2025**

**#7. #1 OR #2 27,662 11 Feb 2025**

**#6. 'osteomyelitis':ab,ti OR 'osteomyelitides':ab,ti 34,436 11 Feb 2025**

**#5. 'osteomyelitis'/exp OR 'osteomyelitis' 62,848 11 Feb 2025**

**#4. 'blood sedimentation':ab,ti OR 'blood 122,069 11 Feb 2025**

**sedimentation rate':ab,ti OR 'sedimentation rate,**

**erythrocyte':ab,ti OR 'erythrocyte sedimentation**

**rate':ab,ti OR 'esr':ab,ti OR 'inflammatory**

**marker':ab,ti OR 'inflammatory markers':ab,ti**

**#3. 'erythrocyte sedimentation rate'/exp OR 79,698 11 Feb 2025**

**'erythrocyte sedimentation rate'**

**#2. 'diabetic feet':ab,ti OR 'diabetic foot 18,752 11 Feb 2025**

**disease':ab,ti OR 'diabetic foot syndrome':ab,ti**

**OR 'diabetic foot':ab,ti**

**#1. 'diabetic foot'/exp OR 'diabetic foot' 27,607 11 Feb 2025**

****Cochrane: 13****

Search Name:

Date Run: 11/02/2025 15:05:19

Comment:

ID Search Hits

#1 MeSH descriptor: [Diabetic Foot] explode all trees 1612

#2 (Foot Ulcer, Diabetic ):ti,ab,kw or (Feet, Diabetic):ti,ab,kw or (Foot, Diabetic):ti,ab,kw or (Diabetic Feet):ti,ab,kw 4738

#3 MeSH descriptor: [Blood Sedimentation] explode all trees 610

#4 (Rates, Erythrocyte Sedimentation):ti,ab,kw or (Erythrocyte Sedimentation Rate):ti,ab,kw or (Erythrocyte Sedimentation Rates):ti,ab,kw or (Rate, Erythrocyte Sedimentation):ti,ab,kw or (Sedimentation Rate, Erythrocyte):ti,ab,kw or (Sedimentation Rates, Erythrocyte):ti,ab,kw or (Erythrocyte Sedimentation):ti,ab,kw or ( Sedimentation, Blood):ti,ab,kw or (Sedimentation, Erythrocyte):ti,ab,kw or (ESR)(inflammatory marker):ti,ab,kw or (inflammatory markers):ti,ab,kw 21550

#5 MeSH descriptor: [Osteomyelitis] explode all trees 210

#6 (Osteomyelitis):ti,ab,kw or (Osteomyelitides):ti,ab,kw 748

#7 #1 OR #2 4738

#8 #3 OR #4 21550

#9 #5 OR #6 755

#10 #7 AND #8 AND #9 13

**Wanfang Data: 21**

(（糖尿病足 and 骨髓炎） and （红细胞沉降率 or ESR or 炎症标记物）)

**OVID: 34**

(“erythrocyte sedimentation rate” OR “inflammatory marker”OR “ESR”) AND “diabetic foot osteomyelitis”
